# 1527. Use of a SARS-CoV-2 Strand-specific Assay to detect Relapse or Ongoing Infection at a Tertiary Care Center, California 2020–2022

**DOI:** 10.1093/ofid/ofac492.089

**Published:** 2022-12-15

**Authors:** Gustavo A Contreras Anez, Jessica Ferguson, Caitlin A Contag, Lucy S Tompkins, John Shepard, Ayelet Rosenthal, Aruna Subramanian, Benjamin A Pinsky, Jorge Salinas

**Affiliations:** Stanford University, Stanford, CA; Stanford University, Stanford, CA; Stanford University, Stanford, CA; Stanford University, Stanford, CA; Stanford University, Stanford, CA; Stanford University, Stanford, CA; Stanford University, Stanford, CA; Stanford University, Stanford, CA; Stanford University, Stanford, CA

## Abstract

**Background:**

Determining if a patient with severe acute respiratory syndrome coronavirus 2 (SARS-CoV-2) remains infectious can be challenging in patients with severe disease or those profoundly immunosuppressed. We use an assay that detects minus-strand RNA as a surrogate for actively replicating SARS-CoV-2. Here, we describe a cohort of patients who had strand-assay reversion: a detectable minus strand-specific assay after having had an undetectable value which may signify relapse of infection or reinfection.

**Methods:**

We used a 2-step rRT-PCR specific to the minus strand of the SARS-CoV-2 envelope gene. The strand-specific assay is used to evaluate for infectivity in asymptomatic patients with a positive screening admission or pre-procedural test or in cases when ongoing infection was suspected (critical illness or profound immunosuppression). We collected minus strand-specific assays performed at Stanford Healthcare during August 2020–April 2022. We describe basic demographics and clinical characteristics for patients who reverted from undetectable to detectable using the minus strand-specific assay.

**Results:**

A total of 2,505 strand-specific tests were collected from August 2020–April 2022 from 2,064 patients. A total of 292 (14%) had two or more strand-specific tests. Of them, 19 (7%) had an undetected minus strand-specific assay followed by a subsequent detectable value. Of them, seven (37%) had a minus strand-specific CT value of < 33. Most were male (n=4), median age was 54 (range: 8–62). All were profoundly immunosuppressed: Four had hematologic malignancies and three were post transplantation (kidney, lung, bone marrow). Median time from onset of symptoms or first positive test to reversion was 41 days (range:27–159). Median time from undetectable to detectable minus strand specific test was 26 days (range:4–34). All cases were considered relapses or ongoing infection rather than a new infection.

**Conclusion:**

Among patients with SARS-CoV-2 infection and consecutive strand-specific testing, a small proportion reverted from negative to positive. Most were profoundly immunosuppressed. The strand-specific assay can be an important tool in the evaluation of suspected cases of relapse or reinfection.

**Disclosures:**

**Aruna Subramanian, MD**, Gilead Sciences: Grant/Research Support|Regeneron, Inc: Grant/Research Support.

